# The psychosocial impact of climate change: an umbrella review and meta-analysis

**DOI:** 10.3389/fpubh.2026.1899420

**Published:** 2026-07-09

**Authors:** Marcelo Leiva-Bianchi, Francisco Ahumada-Méndez, Cristian Cáceres, Carlos Serrano, Kenny Díaz-Arcaño

**Affiliations:** 1 Laboratory of Methodology, Behavior and Neuroscience, Faculty of Psychology, Universidad de Talca, Talca, Chile; 2 Department of Psychology, Faculty of Health Sciences, Universidad Católica del Maule, Curicó, Chile; 3 Faculty of Social Sciences and Humanities, Universidad Autónoma de Chile, Talca, Chile

**Keywords:** climate change, mental health, meta-analysis, psychosocial impact, systematic review, umbrella review

## Abstract

Climate change is increasingly recognized as a psychosocial and mental health challenge, yet the evidence remains fragmented across exposure types, populations, and outcome definitions. This umbrella review and meta-analysis synthesized evidence on the psychosocial impact of climate change and examined the usefulness of the Psychosocial Impact Model (PSIM) as an interpretive framework. Systematic searches were conducted in PubMed, Scopus, and Web of Science following PRISMA guidelines. Nine systematic reviews comprising 345 primary studies were included, and methodological quality was assessed with AMSTAR 2. Random-effects meta-analyses were conducted when sufficient quantitative data were available from high-quality reviews. Findings showed that climate change was associated with psychosocial variables across three exposure contexts: (1) acute, (2) chronic, and (3) symbolic. Outcomes included: psychological distress, depressive symptoms, posttraumatic stress, solastalgia, ecological grief, and climate anxiety, alongside adaptive responses such as resilience, posttraumatic growth, and community cohesion. Meta-analyses showed positive associations between climate-related anxiety or worry and mental health symptoms, with pooled correlations ranging from small to moderate magnitude (*r* = 0.17 to 0.37). The strongest associations were observed for climate anxiety with depressive symptoms (*r* = 0.37, 95% PI [0.14, 0.61], *p* < 0.001, *I*^2^ = 92%) and stress (*r* = 0.35, 95% PI [−0.01, 0.71], *p* < 0.001, *I*^2^ = 94%). Wide prediction intervals indicated substantial between-study variability; pooled estimates should be interpreted cautiously as summaries of heterogeneous associations. Overall, direct exposure to climate-related events and perceived climate risk were associated with psychosocial distress, supporting the value of PSIM as an integrative framework.

**Systematic Review Registration:**

https://osf.io/64rng, OSF.

## 1 Introduction

Climate change is one of the greatest challenges of the 21st century and affects all of humanity (1). Rising greenhouse gas emissions have contributed to extreme events such as droughts, floods, hurricanes, wildfires, and heat and cold waves (2), generating material losses, social disruption, and recurrent exposure to potentially traumatic conditions (2, 3). Although these impacts are global in scope, they are unevenly distributed and disproportionately affect socioeconomically vulnerable populations, as well as individuals with preexisting mental health conditions (4–7). In this context, both direct exposure to extreme climate-related events and the persistent perception of climate threat have been associated with relevant psychosocial consequences (8). This is particularly relevant in settings marked by structural vulnerability and repeated exposure (9, 10).

The concept of psychosocial impact was initially developed in the study of large-scale traumatic events, emphasizing that the same exposure may give rise to both adaptive and disruptive responses (11). Since then, it has been extended to diverse traumatic contexts, including disasters and climate change. From this perspective, psychosocial impact refers to the set of psychological and social effects that an event generates in individuals and communities as a function of exposure, vulnerability, and protective resources (12–14). This framework is especially relevant for the study of stressful and potentially traumatic phenomena because it allows the integration of disruption and adaptation within a single conceptual lens.

From this perspective, responses to potentially traumatic events may be disruptive or resilient. This includes those intensified by climate change (9, 15, 16). To account for this variability, the Psychosocial Impact of Disasters Model [PSIM; (13, 14, 17, 18)] integrates event-related vulnerability, personal beliefs, and post-event responses. It distinguishes different impact profiles, including traumatic and resilient responses. The model helps address debates on heterogeneous trauma prevalence estimates and divergent post-disaster trajectories (19–21). PSIM provides an interpretive framework for understanding psychosocial outcomes and offers a useful conceptual basis for examining the effects of climate change across diverse contexts of vulnerability (see Supplementary Figure S1). In the present review, this model is used as an interpretive framework to organize and contrast evidence across different exposure contexts, rather than as a predictive model subjected to direct empirical testing.

Psychosocial responses to climate-related events may generate long-term mental health consequences that do not necessarily emerge immediately and may persist or intensify over time (15). In the context of climate change, the available evidence points to a broad spectrum of outcomes, ranging from subclinical emotional distress to clinically significant symptomatology (9, 22). Reported manifestations include posttraumatic stress disorder, anxiety, depression, and suicidal ideation (6, 23, 24), as well as psychosomatic disorders (25), vicarious trauma, worry, and fear (23), poor sleep quality, domestic violence, and alcohol and substance use (22), attachment-related disturbances (6), acute stress reactions, and a diminished sense of self and identity associated with displacement from homes and communities (26). Taken together, these findings suggest that climate change should not be understood only in terms of acute emotional reactions, but also as a source of psychosocial processes with traumatic potential that may activate trajectories of sustained psychological disruption.

Despite the growth of this literature, the relationship between climate change and mental health continues to present important conceptual and methodological challenges. A central difficulty lies in the heterogeneity of what is measured and how the impact of climate change is operationalized, including its trauma-related manifestations (25). In this context, recent studies have incorporated emerging constructs such as eco-anxiety, solastalgia, and ecological grief to characterize psychological responses linked to environmental degradation, reflecting both the expansion of the field and the persistence of conceptual heterogeneity (9, 27). Although much of this literature has focused primarily on mental health outcomes, these studies also illuminate psychological responses that are intrinsic to psychosocial impact. In turn, these responses, encompassing both adaptive and disruptive trajectories, show how individuals’ reactions to climate-related events are dynamically situated within, and reciprocally influenced by broader social and environmental contexts. This interdependence between psychological and social dimensions reinforces the relevance of the psychosocial perspective adopted in this review.

At the same time, cumulative inference remains difficult because existing syntheses often focus on specific exposure types, typically extreme events, or particular populations, and they vary in how psychosocial outcomes are defined and operationalized. As a result, it remains unclear whether the pattern of psychosocial impact differs systematically across acute, chronic, and symbolic climate-related exposures and across contexts of structural vulnerability. Research on the psychosocial impact of climate change is therefore both emerging and highly relevant, yet most studies to date have emphasized mental health outcomes rather than the broader construct of psychosocial impact, which integrates psychological and social effects.

Recent work published in *Frontiers in Public Health* has underscored the relevance of examining climate-related crises through the intersecting lenses of mental health, environmental inequality, disaster response, and community resilience. These contributions include research on the unequal mental health consequences of climate-related disasters, eco-anxiety in contexts of environmental injustice, review-based syntheses of climate change and mental health, quantitative evidence on climate-related suicide risk, and community-based approaches to resilience-building in vulnerable urban settings (28–32). Taken together, this emerging evidence shows that climate-related threats affect not only individual well-being, but also the social and collective capacities that enable communities to interpret, endure, and respond to adversity.

However, despite the increasing number of reviews on climate change and mental health, important limitations remain in the available syntheses. Existing reviews have generally focused on specific exposure types, particular populations, or selected mental health outcomes, which limits the possibility of comparing findings across acute, chronic, and symbolic forms of climate-related exposure. Moreover, the literature has predominantly emphasized mental health outcomes, whereas fewer syntheses have examined these findings within a broader psychosocial framework that integrates both disruptive and adaptive responses at individual and community levels. An umbrella review is therefore warranted at this stage to integrate the growing but dispersed review-level evidence, assess its methodological quality, and clarify how psychosocial impacts vary across exposure contexts and vulnerable populations.

In this review, acute, chronic, and symbolic exposures were used as a conceptual-analytical classification to organize heterogeneous climate-related evidence. Acute exposures refer to identifiable, sudden-onset extreme events, such as floods, wildfires, hurricanes, typhoons, and heat waves. Chronic exposures refer to progressive or sustained environmental changes, such as persistent drought, deforestation, and sea-level rise. Symbolic exposures refer to indirect or anticipatory forms of climate-related exposure mediated by awareness, perception, worry, or concern about climate change. This classification was not intended as a fixed taxonomy, but rather as a pragmatic framework for comparing psychosocial impacts across different modes of climate-related exposure.

Accordingly, this umbrella review provides an integrative synthesis of the psychosocial impact of climate change, including acute exposures, progressive climate-related changes, and symbolic exposure through the perception and awareness of climate risk. The findings have implications for research, clinical practice, and mental health policy. Against this backdrop of fragmented evidence, the present umbrella review aims to evaluate and synthesize the existing evidence on the psychosocial impact of climate change in human populations. Specifically, it examines how psychosocial impact manifests across different vulnerability contexts, explores the extent to which the PSIM helps interpret these effects, and identifies the populations most consistently reported as vulnerable in the available evidence.

## 2 Methods

This umbrella review was conducted in accordance with the Preferred Reporting Items for Systematic Reviews and Meta-Analyses (PRISMA) guidelines (33). The review protocol was preregistered in the Open Science Framework (OSF; osf.io/64rng).

### 2.1 Search strategy

Systematic searches were conducted in PubMed, Scopus, and Web of Science in January 2025. The search strategy combined terms related to climate change and psychosocial impact. The full search strategy is provided in Supplementary material.

### 2.2 Eligibility criteria

Systematic reviews, with or without meta-analysis, that examined the psychosocial impact of exposure to climate change in human populations were eligible for inclusion. Inclusion and exclusion criteria were defined *a priori* and are presented in Supplementary Table S1.

### 2.3 Study selection

Two authors (KD-A and FA-M) independently screened records at the title/abstract and full-text stages. Disagreements were resolved through discussion and consensus. When consensus could not be reached, a third author (ML-B or CC) adjudicated.

### 2.4 Quality assessment

The methodological quality of the systematic reviews included in the umbrella review was assessed using the AMSTAR 2 tool (34). This appraisal was conducted to provide a standardized evaluation of the methodological robustness of the included reviews and to support the interpretation of the synthesized evidence.

### 2.5 Data extraction and descriptive synthesis

Two reviewers independently extracted data using a standardized form. Extracted information included review identification (authors, year), number of primary studies included, geographic location(s), population(s) and target groups, and total sample size when reported, as well as the type of climate exposure (acute, chronic, or symbolic) and the psychosocial or mental health outcomes assessed. When total sample size was not reported in the original review, this was coded as “not reported”.

The extracted findings were synthesized descriptively through structured tables and narrative summaries. Descriptive characteristics of the included reviews, including type of exposure, psychosocial impact, and vulnerable populations, were summarized in the Results section. Detailed characteristics of the included reviews are presented in the Supplementary material. Overlap of primary studies across reviews was examined and documented in order to inform interpretation and guide de-duplication procedures for the meta-analyses.

### 2.6 Meta-analysis

Meta-analyses were conducted using primary studies identified within the three systematic reviews classified as high quality according to AMSTAR 2. No additional stand-alone search for primary studies was performed for this stage. Instead, quantitative information was extracted from those reviews when sufficient data were available to compute a common effect size. Effect sizes were harmonized by converting eligible associations to Pearson’s correlation coefficients (r) when necessary. Correlations were then transformed using Fisher’s r-to-z transformation for analysis and back-transformed to r for reporting. Only conceptually comparable exposure–outcome pairs were pooled. Given the expected heterogeneity across populations, countries, exposure definitions, and outcome measures, random-effects models were used. Prediction intervals were reported alongside pooled estimates to reflect the expected range of effects in comparable future settings. Accordingly, pooled estimates were interpreted as average associations across heterogeneous studies rather than as stable or uniform effects.

The meta-analyses should be interpreted as secondary quantitative syntheses derived from primary studies identified within high-quality systematic reviews rather than as *de novo* systematic reviews of primary studies. Consequently, the quantitative synthesis was dependent on the scope and reporting characteristics of the reviews included in the umbrella review.

Random-effects models were estimated using correlation coefficients transformed with Fisher’s *r*-to-*z* transformation and restricted maximum likelihood (REML) estimation of tau^2^. Heterogeneity was assessed using I^2^, Cochran’s Q, tau, and H^2^. Publication bias was explored using Begg’s and Egger’s tests, fail-safe *N*, and funnel plots. All analyses were conducted in Jamovi using the MAJOR module. In total, eight meta-analyses were performed.

To minimize data dependency, primary studies that appeared in more than one review were identified and included only once. Although this procedure reduced redundancy, it does not eliminate all sources of dependence inherent to secondary synthesis.

Moderator or subgroup analyses were not conducted because the number of studies included in each meta-analysis was small and study-level characteristics such as age, sex, country, and population type were not reported consistently enough across primary studies to support reliable stratified analyses.

## 3 Results

### 3.1 Literature search and study selection

The database search identified 152 records (Web of Science, *n* = 74; Scopus, *n* = 36; PubMed, *n* = 42). After the removal of 68 duplicates, 84 records were screened at the title and abstract level, and 53 were excluded. Full texts were assessed for 31 reports, of which 22 were excluded, resulting in nine systematic reviews included in the umbrella review. The study selection process is presented in the PRISMA flow diagram (Supplementary Figure S2).

### 3.2 Characteristics of the included systematic reviews

The included systematic reviews showed marked methodological and thematic heterogeneity in exposure definitions, outcome operationalization, study populations, and geographic settings. None of the included reviews reported a meta-analysis. Consequently, the quantitative synthesis conducted in the present study was limited to primary studies with extractable data identified within high-quality reviews.

Two reviews explicitly reported reasons for not conducting meta-analysis, noting that, in this emerging field, the heterogeneity of study designs, populations, and assessed variables hindered statistical synthesis (35), whereas in other cases the limited availability of comparable quantitative data made meta-analysis unfeasible (36).

Nevertheless, in three methodologically high-quality reviews, primary studies with sufficient quantitative information were identified to allow statistical synthesis. Based on these studies, the present work conducted meta-analyses. Duplicate studies across reviews were identified and excluded to avoid the inclusion of redundant evidence.

Detailed characteristics of the included reviews are presented in Supplementary Tables S2, S3.

### 3.3 Quality assessment of the included reviews

The nine included systematic reviews showed a heterogeneous distribution in methodological quality, as assessed using the AMSTAR 2 tool. Four reviews (44.4%) were classified as critically low quality, two (22.2%) as moderate quality, and three (33.4%) as high quality (Supplementary Table S4). Given this distribution, findings from high-quality reviews were prioritized in a dedicated synthesis, whereas the full body of included reviews was used primarily for descriptive mapping. Reviews rated as critically low were retained in the descriptive synthesis to preserve a comprehensive map of the available review-level evidence, particularly regarding exposure types, populations, and psychosocial outcomes that remain unevenly represented in the literature. However, these reviews were not used to support the quantitative synthesis, and their findings were interpreted with caution according to their AMSTAR 2 ratings.

### 3.4 Descriptive synthesis of exposures, populations, and psychosocial impacts

#### 3.4.1 Types of climate change exposure

The included reviews addressed three main forms of climate change exposure: acute exposure to extreme climate events (e.g., floods, wildfires, hurricanes, typhoons, and heat waves), chronic exposure associated with progressive environmental processes (e.g., persistent droughts, deforestation, and sea-level rise), and symbolic exposure linked to awareness, perception, and anticipation of climate change. Most reviews included more than one type of exposure (Supplementary Table S3). This exposure-based classification was used to structure the descriptive synthesis and to contextualize the meta-analytic findings.

#### 3.4.2 Participants in the included studies

The included systematic reviews encompassed diverse populations affected by climate change, including disaster-exposed groups, structurally vulnerable communities, and populations experiencing symbolic exposure through climate awareness and risk perception. Among the most frequently studied populations were individuals exposed to natural disasters, Indigenous communities, rural populations, university students, women, children and adolescents, older adults, and individuals with preexisting mental health conditions. Specific groups also included displaced persons, farmers, fishers, coastal communities, emergency personnel, and health professionals (Supplementary Table S2).

#### 3.4.3 Psychosocial impact across reviews

Across reviews, outcomes clustered into trauma-related and affective symptoms, risk and interpersonal harms (e.g., suicidality and violence), and adaptive responses (e.g., resilience and growth). Reviews focusing on extreme events reported that vulnerable populations experienced posttraumatic stress disorder, depression, anxiety, sleep disorders, and eco-anxiety, particularly women, children, older adults, and individuals with preexisting mental health conditions (37). These reviews also described exacerbation of preexisting disorders, increased domestic violence, and adaptive manifestations such as resilience, posttraumatic growth, and enhanced community cohesion (37). In contexts characterized by limited access to resources and protection, anxiety, depression, solastalgia, chronic stress, increased risk of suicide and self-harm, deterioration in emotional well-being, social isolation, and loss of cultural and spiritual identity were reported (38).

Chronic exposure to climate change was associated with ecological grief, loss of cultural identity, interpersonal conflicts, food insecurity, and concerns about the future, which were also linked to increased substance use and domestic violence (39). Reviews addressing symbolic exposure highlighted that environmental change and climate awareness were associated with anxiety, depression, eco-anxiety, stress, substance use, suicidal ideation, and reduced emotional well-being across diverse populations (35, 40, 41).

Other reviews included both acute and chronic exposures. Their findings described declines in mental well-being, including sadness, worry, depression, anxiety, posttraumatic stress, fear, and emotional distress (25, 36, 37, 42), as well as aggressive behaviors, domestic violence, substance use, and suicidal ideation (25, 42). In addition, concern about the future was associated with eco-anxiety, solastalgia, and cognitive impairment in children and older adults (25), as well as with sleep difficulties and distracted thinking (36). Feelings of helplessness, hopelessness, climate anxiety, and solastalgia were also reported (41).

A detailed overview of psychosocial outcomes reported across the included reviews is presented in Supplementary Table S3.

### 3.5 Findings from high-quality reviews

To identify the most methodologically robust findings, a specific analysis was conducted of the three systematic reviews classified as high quality according to AMSTAR 2 (35, 37, 40). Together, these three high-quality reviews comprised 77 of the 345 primary studies covered by the nine systematic reviews included in the umbrella review, representing 22.3% of the total review-level evidence base. The remaining six reviews were retained for the descriptive synthesis but were not included in the quantitative synthesis because they were classified as moderate or critically low quality according to AMSTAR 2 and/or did not provide sufficient extractable quantitative data to compute comparable effect sizes. This decision was made to ensure that the meta-analytic estimates were based on the most methodologically robust and quantitatively usable evidence available. Based on the primary studies included in these three high-quality reviews, and when sufficient quantitative data were available, eight meta-analyses were conducted.

Across the eight meta-analyses, heterogeneity was very high (*I*^2^ > 90%). Therefore, the pooled correlations should be interpreted as average associations across heterogeneous studies rather than as stable or uniform effects. Prediction intervals indicated that the magnitude of the associations may vary substantially across contexts, with several intervals including the null value.

#### 3.5.1 Types of exposure

Tito et al. (37) examined combined exposure, including both acute and chronic forms, such as typhoons, floods, sea-level rise, and droughts. In contrast, Gianfredi et al. (40) and Cosh et al. (35) focused on symbolic exposure linked to awareness and perception of climate change, even in the absence of direct exposure to extreme events.

#### 3.5.2 Psychosocial impact

The three high-quality systematic reviews reported a broad spectrum of psychosocial effects associated with climate change. Among the adverse outcomes, clinical conditions such as depression, anxiety, posttraumatic stress disorder, suicidal ideation, and adjustment disorders were identified (35, 37, 40). In the case of symbolic exposure, symptoms such as eco-anxiety, climate distress, persistent hopelessness, and psychological distress were reported even in the absence of direct exposure to extreme climate events (35, 40).

Tito et al. (37) also reported consequences associated with environmental degradation, including domestic violence, exacerbation of preexisting mental disorders, problematic substance use, food insecurity, interpersonal conflicts, declines in subjective well-being, and changes in culture and identity.

Alongside these adverse effects, adaptive responses were also identified. Specifically, Tito et al. (37) reported resilience, posttraumatic growth, and increased community cohesion as psychosocial responses observed in some exposed populations.

#### 3.5.3 Vulnerable populations

Tito et al. (37) identified women, older adults, rural and Indigenous communities, displaced persons, and individuals with preexisting mental health conditions as particularly vulnerable populations. Gianfredi et al. (40) and Cosh et al. (35) emphasized impacts among young adults and university students, particularly in relation to eco-anxiety and uncertainty about the future climate. Tito et al. (37) also reported psychosocial risk among health professionals and emergency personnel exposed to extreme events.

### 3.6 Meta-analysis results

Based on the primary studies included in the three high-quality systematic reviews, eight meta-analyses were conducted using random-effects models to estimate associations between different manifestations of the psychosocial impact of climate change. Pooled effect size estimates, prediction intervals, and heterogeneity statistics are summarized in Table 1. Overall, pooled correlations ranged from small to moderate in magnitude. However, all meta-analyses showed very high heterogeneity (*I*^2^ > 90%), indicating that pooled estimates should be interpreted as average associations across heterogeneous studies rather than as stable estimates of effect.

**Table 1:** Summary of meta-analytic results on psychosocial impacts of climate change.

Association	*k*	Effect size (*r*)	95% PI	*p*	*I*^2^ (%)
Climate anxiety - depressive symptoms	6	0.37	[0.14, 0.61]	< 0.001	92
Climate anxiety - stress	6	0.35	[−0.01, 0.71]	< 0.001	94
Climate worry - anxiety	6	0.20	[−0.21, 0.61]	0.013	96
Climate worry - stress	6	0.26	[−0.03, 0.55]	< 0.001	93
Climate worry - pro-environmental behaviors	3	0.30	[−0.12, 0.71]	0.007	96
Climate worry - depressive symptoms	8	0.22	[−0.21, 0.64]	0.003	99
Climate knowledge - anxiety	6	0.17	[−0.20, 0.54]	0.022	92
Exposure to typhoon Haiyan - psychological distress	4	0.23	[−0.27, 0.72]	0.052	91

*r*, correlation coefficient (Fisher’s *r*-to-*z* transformed and back-transformed for reporting); *k*, number of primary studies; PI, prediction interval. Prediction intervals are reported due to substantial heterogeneity across studies.

Regarding climate anxiety and mental health, the meta-analyses showed positive and statistically significant associations between climate anxiety and depressive symptoms (*r* = 0.37, *p* < 0.001; Supplementary Figure S3) and between climate anxiety and stress (*r* = 0.35, *p* < 0.001; Supplementary Figure S4). In both cases, heterogeneity was high (*I*^2^ > 90%). Influence analyses did not identify influential studies. Publication bias was explored using standard methods, and given the small number of studies included in each meta-analysis, these results were interpreted cautiously. Prediction intervals indicated substantial variability in the expected range of effects across comparable settings, with the interval for climate anxiety and stress including the null value.

Similarly, positive and statistically significant associations were observed between climate worry and anxiety (*r* = 0.20, *p* = 0.013; Supplementary Figure S5) and between climate worry and stress (*r* = 0.26, *p* < 0.001; Supplementary Figure S6), both with high heterogeneity. Climate worry was also significantly associated with pro-environmental behaviors (*r* = 0.30, *p* = 0.007; Supplementary Figure S7) and depressive symptoms (*r* = 0.22, *p* = 0.003; Supplementary Figure S8). The meta-analysis examining the association between climate worry and depressive symptoms showed very high heterogeneity (*I*^2^ > 98%). Although the pooled estimate was statistically significant, the prediction interval included the null value, indicating substantial uncertainty regarding the expected effect in comparable future settings. This result should therefore be interpreted as an average association across highly heterogeneous studies rather than as a stable effect estimate.

In addition, a positive and statistically significant correlation of small magnitude was identified between climate knowledge and anxiety (*r* = 0.17, *p* = 0.022; Supplementary Figure S9). Heterogeneity among the included studies was high (*I*^2^ = 92.2%), and publication bias was explored using standard methods and interpreted cautiously given the small number of studies. The prediction interval included the null value, suggesting that the expected association may vary substantially across contexts.

Finally, the meta-analysis evaluating the association between exposure to Typhoon Haiyan and psychological distress did not reach statistical significance (*r* = 0.23, *p* = 0.052; Supplementary Figure S10), and heterogeneity was very high (*I*^2^ = 91.3%). The prediction interval was wide and included the null value, indicating substantial uncertainty in the expected range of effects across comparable settings. Given the small number of studies included in this analysis (*k* = 4), this pooled estimate should be interpreted as an exploratory average association and should not be considered evidence of a statistically significant or stable effect.

## 4 Discussion

The findings of this umbrella review, based on nine systematic reviews encompassing 345 primary studies, indicate that climate change is associated with relevant psychosocial impacts across diverse human populations. These impacts emerge through different forms of exposure (acute, chronic, and symbolic) and range from subclinical emotional distress to clinically significant psychopathological outcomes.

The meta-analyses conducted in the present study provide exploratory quantitative summaries that complement the descriptive syntheses derived from the included reviews. Taken together, the pooled estimates suggest small-to-moderate average associations between climate anxiety, climate worry, and indicators of psychological distress, including depressive symptoms, anxiety, and stress. However, heterogeneity was very high across all meta-analyses, indicating substantial variability across studies, populations, exposure definitions, and outcome measures. Accordingly, these pooled estimates should not be interpreted as stable or uniform effects, but as average associations within a heterogeneous evidence base. Importantly, because the meta-analytic findings were based on correlational data, they should be interpreted as associations rather than causal or directional relationships. The prediction intervals further indicate that the magnitude of these associations may vary substantially across comparable future settings, with several intervals including the null value. The positive association between climate worry and pro-environmental behaviors also suggests that climate-related distress may be linked to behavioral responses oriented toward adaptation or mitigation. By contrast, the association between exposure to Typhoon Haiyan and psychological distress did not reach statistical significance. Given the small number of studies included in this meta-analysis (*k* = 4), the very high heterogeneity, and the wide prediction interval including the null value, this result should be interpreted cautiously and not as evidence of a stable association.

The descriptive evidence synthesized in this review further shows that the psychosocial impact of climate change is not homogeneous, but varies according to the type of exposure. Acute exposure to extreme climate events is associated with increased levels of posttraumatic stress disorder, anxiety, depression, sleep disorders, and domestic violence, particularly in structurally vulnerable populations (37, 38). In turn, chronic exposure derived from progressive environmental changes is linked to more persistent and contextually embedded outcomes, such as ecological grief, solastalgia, loss of cultural identity, and food insecurity (39, 42). Symbolic exposure, understood as awareness and anticipation of environmental degradation, is also associated with psychological distress, including eco-anxiety, persistent worry, depressive symptoms, and stress, even in the absence of direct exposure to extreme events (35, 40, 41). Rather than providing precise estimates of stable effect magnitudes, the meta-analytic findings should therefore be interpreted as exploratory summaries of average associations between climate-related psychological processes and mental health indicators, with substantial variability across contexts.

From a conceptual perspective, these findings reflect the growing relevance of emerging psychological constructs, such as eco-anxiety, solastalgia, and ecological grief, in attempts to characterize subjective experiences linked to climate change. Although these concepts are still undergoing theoretical delimitation and empirical validation, their recurrence across the literature suggests that traditional frameworks may be insufficient to capture contemporary forms of distress associated with global, diffuse, and prolonged environmental threats.

The focused analysis of the methodologically high-quality systematic reviews provides a more cautious basis for interpreting these patterns. These reviews suggest that different forms of climate change exposure may activate diverse psychosocial trajectories, including both disruptive and adaptive responses. Among the most frequently reported adverse manifestations are anxiety, depression, posttraumatic stress disorder, suicidal ideation, and adjustment disorders. In addition, emerging expressions of psychological distress, such as eco-anxiety and climate distress, are described particularly among young adults and university students, indicating that the psychosocial impact of climate change is not restricted to those who directly experience extreme events, but also extends to those who anticipate their consequences.

Beyond individual-level manifestations, the reviewed evidence also shows that climate change is associated with social and community-level impacts, including interpersonal stress, domestic violence, food insecurity, and declines in subjective well-being (37). At the same time, some high-quality reviews document adaptive responses, such as resilience, posttraumatic growth, and strengthened community cohesion in specific contexts (37). These findings underscore the complexity of the psychosocial impact of climate change and support the view that disruption and adaptation may coexist rather than operate as mutually exclusive outcomes.

The most vulnerable populations identified in the available evidence include women, older adults, rural and Indigenous communities, displaced persons, and individuals with preexisting mental health conditions. In addition, young adults, university students, and professionals exposed to extreme climate events show elevated levels of psychological distress, particularly in relation to uncertainty about the future climate (35, 37, 40). These patterns reinforce the importance of recognizing the unequal distribution of climate vulnerability in both research and intervention design.

The findings of this review have implications not only for research and clinical practice, but also for the development of public and community mental health policies. Given that psychosocial responses vary according to exposure and vulnerability, mental health strategies may benefit from preventive and supportive approaches that explicitly recognize the interrelation between environmental, psychosocial, and traumatic stress factors. Policies integrating psychosocial risk assessment, equitable access to mental health services, and education on adaptive coping may help guide more context-sensitive and socially responsive interventions.

In this context, the PSIM (13, 14, 17, 18, 43) emerges as a useful theoretical framework for integrating the different findings. The model conceptualizes both acute exposures and chronic, symbolic climate change processes as stressors that may trigger disruption or adaptation trajectories. Outcomes depend on exposure level, available resources, and interpretive frameworks. Current evidence nevertheless indicates the need for further empirical evaluation across diverse climate contexts and longer time frames. This would apply to the trauma trajectories model (19–21, 44), particularly to estimate the prevalence of each trajectory and identify when affected populations shift between impact profiles. Regarding the temporal dimension, these trajectories have been conceptualized as unfolding over extended periods following potentially traumatic events, with more recent formulations avoiding a fixed temporal cutoff (19, 21); (Supplementary Figure S11). Longer durations of six to 7 years have been reported after earthquakes (45).

As a narrative sensitivity check, the main descriptive patterns were re-examined by giving priority to reviews rated as moderate or high quality. This check indicated that the central conclusions regarding the association between climate-related exposures and psychosocial or mental health outcomes were primarily supported by reviews rated as moderate or high quality. Reviews rated as critically low contributed mainly to contextual mapping and were not used to support meta-analytic estimates. Therefore, conclusions based on critically low-quality reviews were treated as tentative and interpreted with caution.

This review has several limitations. First, the search was restricted to systematic reviews with full-text availability, which may have excluded relevant evidence. Second, the methodological heterogeneity of the primary studies and the variability in the quality of the included reviews limit the comparability of findings. Reviews rated as critically low according to AMSTAR 2 were retained only for descriptive mapping, were interpreted with caution, and were not used to support the quantitative synthesis. Although the review adopts a psychosocial framework, the quantitative evidence available in literature remains predominantly focused on mental health outcomes, which limits the empirical assessment of broader social dimensions of psychosocial impact. In addition, the meta-analyses were based on a limited number of studies for some associations, which may have influenced the precision of the estimates and contributed to the observed heterogeneity. This limitation, together with the inconsistent reporting of relevant study-level characteristics, also prevented reliable moderator or subgroup analyses; therefore, potential sources of heterogeneity could not be formally examined. Moreover, the quantitative syntheses were based on correlational data; therefore, the observed associations should not be interpreted as causal relationships or as evidence of directional effects. Likewise, the classification of exposures and impacts was conducted by the authors, which introduces a degree of subjectivity. Finally, because the included reviews did not report their own meta-analyses, the availability of comparable quantitative evidence was limited.

Taken together, the findings of this review support the need to recognize climate change as a relevant psychosocial determinant of mental health, while interpreting the available quantitative estimates with caution due to the high heterogeneity of the evidence. For clinicians, these findings underscore the importance of assessing climate-related distress without pathologizing all forms of climate concern, while identifying cases in which distress is persistent, impairing, or associated with depressive, anxious, traumatic, or suicidal symptoms. For public health professionals, the results point to the need to integrate psychosocial risk assessment, adaptive coping education, referral pathways, and community-based support into climate adaptation and disaster preparedness programs. For policymakers, the evidence highlights the need to incorporate mental health and psychosocial support into climate policy, with attention to equitable access to services and the unequal distribution of climate-related vulnerability. Future research should prioritize longitudinal designs, the validation of specific instruments, and the empirical evaluation of theoretical models capable of explaining both disruptive and adaptive psychosocial responses to climate change.

## Data Availability

The data analyzed in this study is subject to the following licenses/restrictions: the data analyzed in this study were extracted from previously published systematic reviews identified through searches in PubMed, Scopus, and Web of Science. No original data were generated for this article, and no separate dataset has been deposited in a public repository. The review protocol is available at OSF: https://osf.io/64rng/. Additional extracted data supporting the conclusions of this article are available from the corresponding author upon reasonable request. Requests to access these datasets should be directed to KD-A, kenny.diaz@utalca.cl.
