# Supplementary material for: The psychosocial impact of climate change: an umbrella review and meta-analysis
Source: Front Public Health. 2026 Jul 9;14:1899420. doi: 10.3389/fpubh.2026.1899420 (PMC13391497; doi:10.3389/fpubh.2026.1899420)
Supplement: Supplementary file 1 [file Supplementary_File_1.docx]

Supplementary Material

# Supplementary Figures

**Supplementary Figure S1.**

*Psychosocial Impact Model (PSIM).*


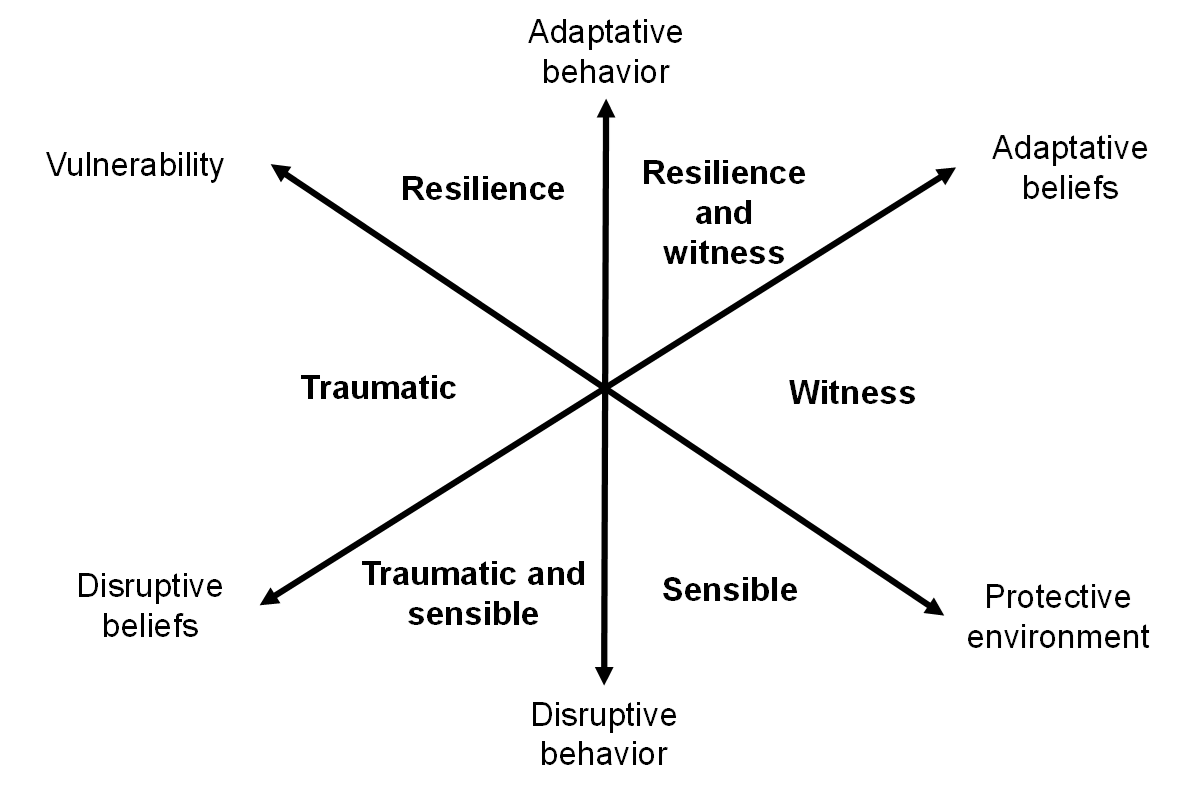


**Supplementary Figure S2.**

*PRISMA Flow Diagram*


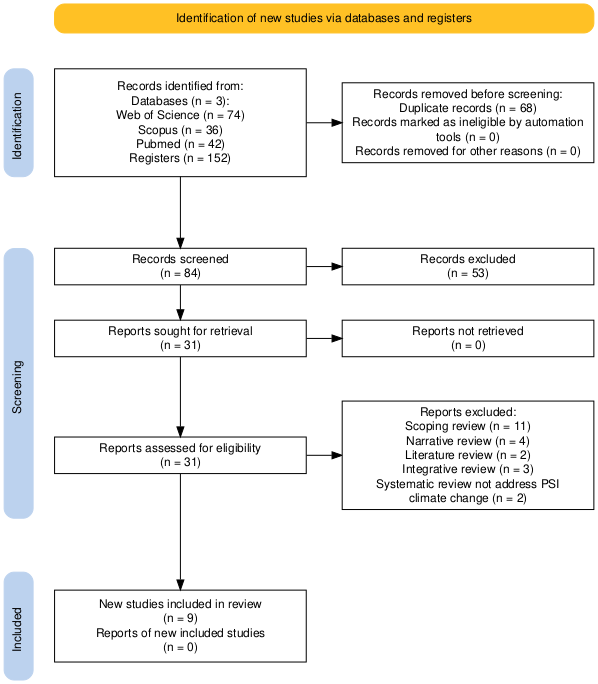


*Note*. PRISMA flow diagram generated using a web-based PRISMA tool.

**Supplementary Figure S3.**

*Forest plot for the meta-analysis examining the association between* *climate anxiety and depressive symptoms*


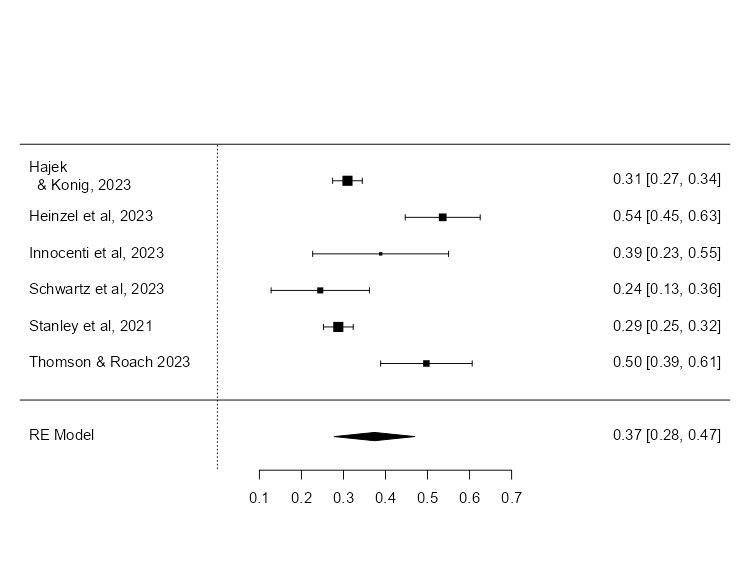


**Supplementary Figure S4.**

*Forest plot* *for the meta-analysis examining the association between climate anxiety and stress.*


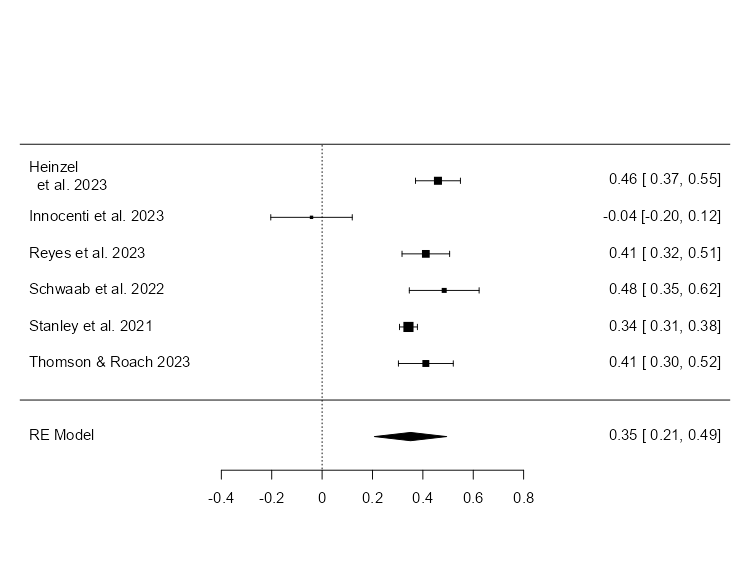


**Supplementary Figure S5.**

*Forest plot for the meta-analysis examining the association between climate worry and anxiety*


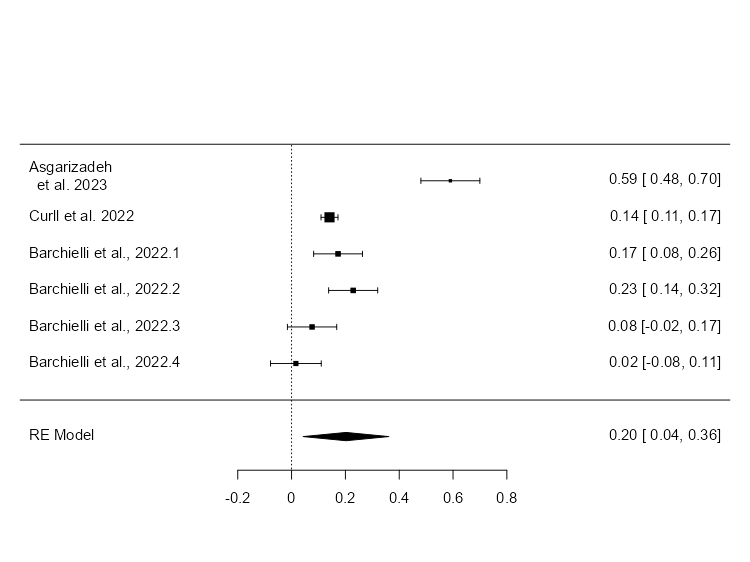


**Supplementary Figure S6.**

*Forest plot for the meta-analysis examining the association between climate worry and stress*


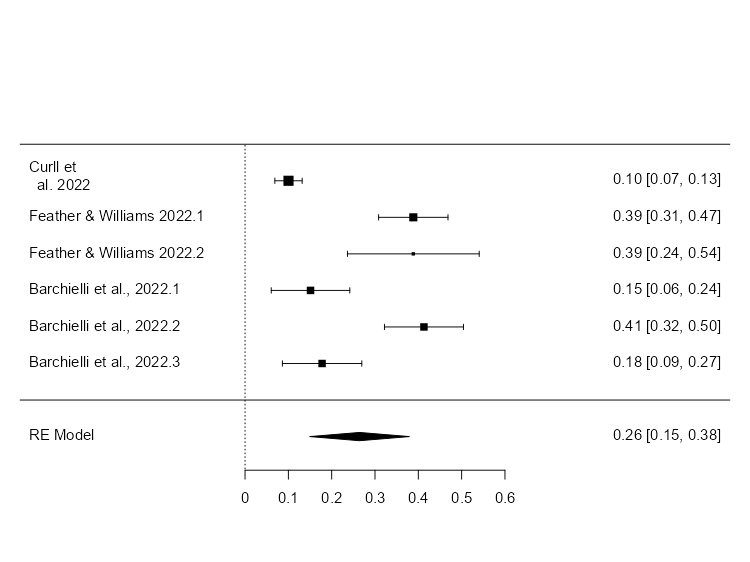


**Supplementary Figure S7.**

*Forest plot for the meta-analysis examining the association between climate worry and pro-environmental behaviors.*
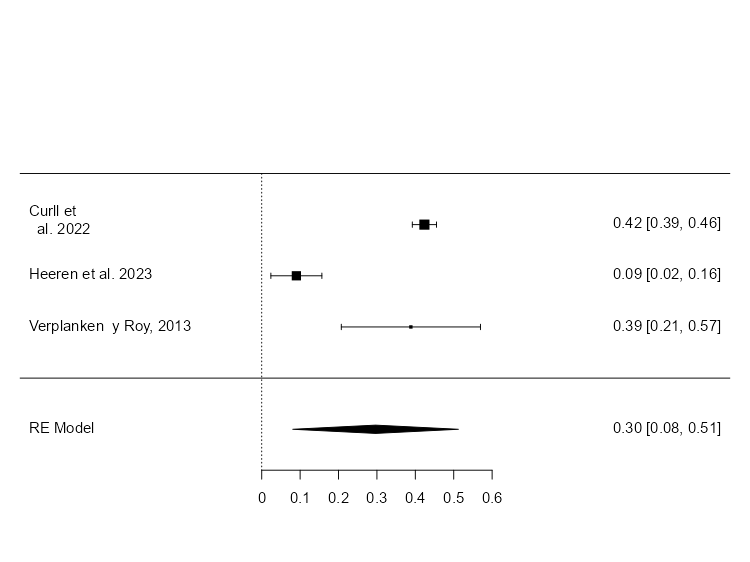


**Supplementary Figure S8.**

*Forest plot for the meta-analysis examining the association between climate worry and depressive symptoms.*


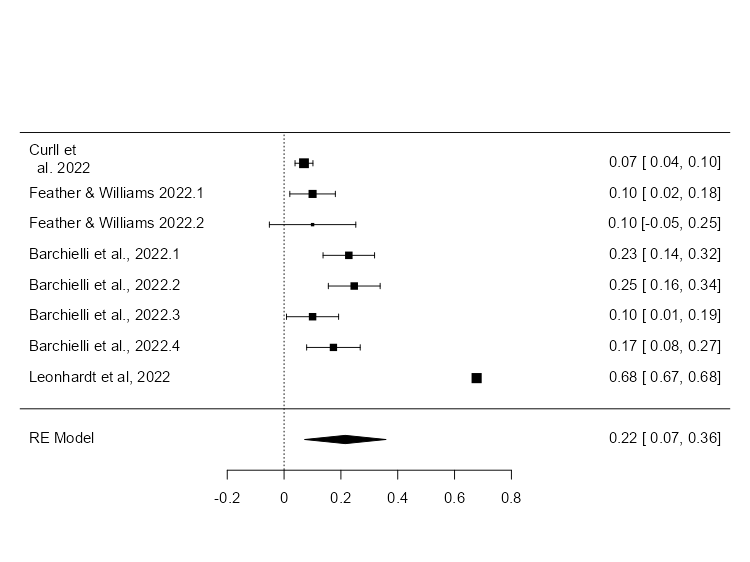


**Supplementary Figure S9.**

*Forest plot for the meta-analysis examining the association between climate knowledge and anxiety.*


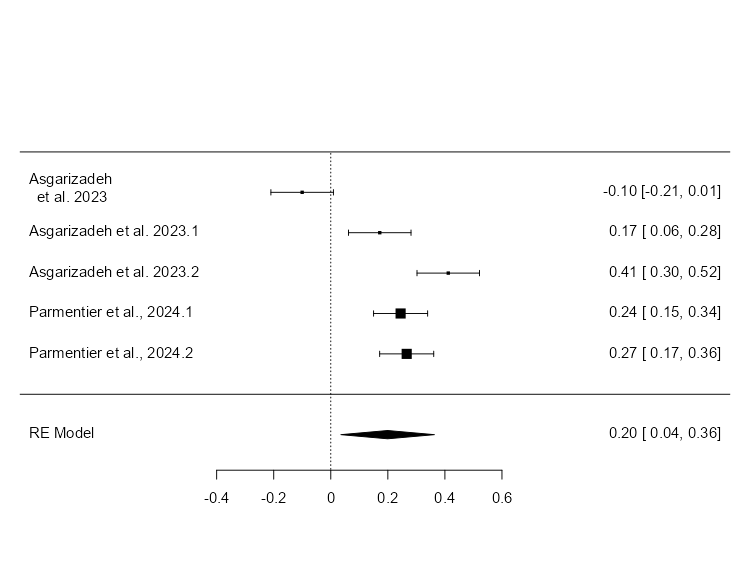


**Supplementary Figure S10.**

*Forest plot for the meta-analysis examining the association between exposure to Typhoon Haiyan and psychological distress.*


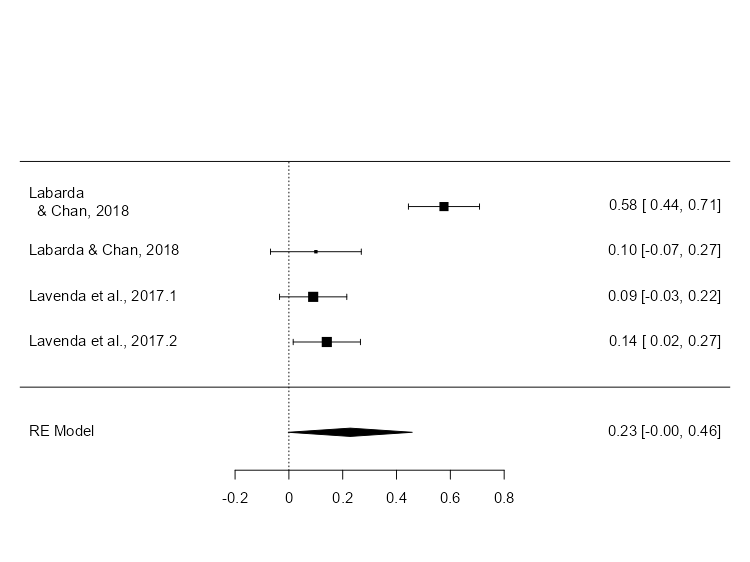


**Supplementary Figure S11.**

*Prototypical outcome trajectories following event.*


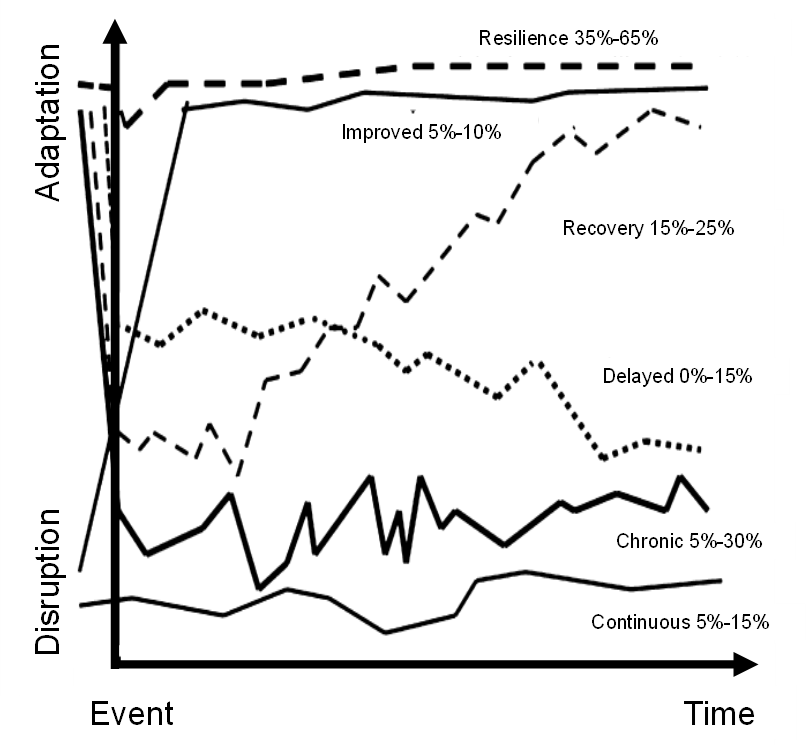


Note: adapted from Bonanno and Diminich (2013) and Bonanno et al. (2023).

# Supplementary Tables

**Supplementary Table S1.**

*Inclusion and Exclusion Criteria*

| **Inclusion Criteria** | **Exclusion Criteria** |
| --- | --- |
| Systematic review articles, with or without meta-analysis. | Scoping reviews, narrative reviews, and literature reviews. |
| Published in peer-reviewed journals. | Book chapters, commentaries, editorials, journalistic texts, theses, and conference abstracts. |
| Examining the psychosocial impact of exposure to climate change in human populations. | Reviews of reviews. |
| Including exclusively human participants exposed to climate change | Reviews that do not address the direct and/or indirect psychosocial impacts of climate change. |
| Full-text availability | Reviews with incomplete or insufficient empirical data. |

**Supplementary Table S2.**

*Characteristics of Included Systematic Reviews: Methodological and Contextual Features*

| Authors (year) | Studies included | Geographic location | Population | Sample size |
| --- | --- | --- | --- | --- |
| Tito et al. (2024) | 32 | Philippines | General population exposed to natural disasters / Specific vulnerable groups (women, children, older adults, individuals with preexisting mental health conditions) / Emergency medical personnel / Displaced persons / University students / Rural and urban communities | Not reported |
| Gianfredi et al. (2024) | 10 | United States, Australia, Canada, Bangladesh, Norway, Italy, Germany | Youth, adults, students, health professionals, Indigenous communities and vulnerable rural populations, general population | 135,468 |
| Lebel et al. (2022) | 26 | Canada, Alaska, Sweden, Russia | Indigenous populations | Not reported |
| Fahrudin et al. (2024) | 35 | Not reported | Individuals with limited access to resources, information, and protection / Groups at risk due to socioeconomic conditions | Not reported |
| Vecchio et al. (2022) | 23 | Canada, Australia, Sweden, Taiwan, Greenland, Fiji, West Africa, Russia | Indigenous populations | Not reported |
| Cianconi et al. (2020) | 163 | Global | Individuals exposed to natural disasters / Women / Children and adolescents / Older adults / Individuals with preexisting mental health conditions / Individuals with low socioeconomic status / Indigenous communities / Relief workers and emergency personnel / Rural and agricultural populations / Urban populations / Migrants and climate-displaced persons | Not reported |
| Cosh et al. (2024) | 35 | Global | University students / Pregnant women / Young adults / Clinical populations / Adults experiencing eco-anxiety / Diverse populations | 45,667 |
| Mengesha and Sarnyai (2025) | 6 | Solomon Islands, Tuvalu, Cook Islands | Residents of rural and coastal communities / General population / Farmers and fishers / Communities at risk of relocation / Individuals with preexisting vulnerabilities (women, older adults, individuals with economic hardship) | Not reported |
| Soutar and Wand (2022) | 15 | United States, Australia, Norway, South Korea, Sweden, Ghana, Tuvalu, Fiji, Cyprus, New Zealand, United Kingdom – 1 study not identified | Young university students / General population / Agricultural producers / Residents of remote communities / Climate scientists and activists / Farmers and livestock producers / Indigenous communities / Corporate sustainability professionals | 1,361 |

**Supplementary Table S3.**

*Characteristics of Included Systematic Reviews: Climate Vulnerability and Outcomes*

| Authors (year) | Vulnerability | Psychosocial impact |
| --- | --- | --- |
| Tito et al. (2024) | Typhoons, floods, sea level rise, droughts | Posttraumatic stress disorder (PTSD), depression, anxiety, sleep disorders, eco-anxiety, resilience, posttraumatic growth, increased community cohesion, domestic violence, exacerbation of preexisting mental health conditions, reduced subjective well-being, changes in culture and identity, food insecurity, stress, and interpersonal conflicts |
| Gianfredi et al. (2024) | Climate change awareness | Depression, anxiety, eco-anxiety, stress, adjustment disorder, substance use, dysphoria, suicidal ideation, reduced well-being, reduced resilience, and lower psychological well-being |
| Lebel et al. (2022) | Changes in ice conditions (reduced thickness, melting, changes in ice formation and breakup), changes in climate (increased temperatures, unpredictable climate patterns, more intense storms), changes in fauna (altered migration, species decline, changes in animal behavior), changes in vegetation (emergence of new species, decline of traditional plants, changes in food quality), changes in water cycles (droughts, floods, changes in water quality), permafrost thaw, and coastal erosion | Sadness, anxiety, fear, frustration, loss of traditional livelihoods, suicidal ideation, problematic substance use, domestic violence and compulsive behaviors, food insecurity, loss of culture, alcohol and drug abuse |
| Fahrudin et al. (2024) | Extreme climate events | Anxiety, depression, solastalgia, posttraumatic stress disorder (PTSD), eco-anxiety, psychological trauma, chronic stress, increased rates of suicide and self-harm, impaired emotional well-being, exacerbation of preexisting mental health conditions, social isolation, anxiety related to economic insecurity and displacement, and loss of cultural and spiritual identity |
| Vecchio et al. (2022) | Floods, cyclones, variability in ice, snow, and rainfall, sea level rise, droughts, extreme temperatures, displacement, loss of connection to the land, and variability in animal and insect populations | Reduced psychological well-being (sadness, worry, fear, emotional distress, frustration, anger, aggression, domestic violence, substance use, suicidal ideation), and depression, anxiety, and posttraumatic stress |
| Cianconi et al. (2020) | Climate change, extreme heat and heatwaves, floods, sea level rise, hurricanes, droughts, wildfires, and deforestation | Posttraumatic stress disorder (PTSD), depression, anxiety, sleep disorders, suicidal ideation, aggressive behaviors, increased substance use, eco-anxiety, ecological grief, solastalgia, biospheric concern, mood disorders, increased violence, adjustment disorders, and dissolution of community bonds |
| Cosh et al. (2024) | Climate change awareness | Eco-anxiety, psychological distress, depressive symptoms, anxiety symptoms, and posttraumatic stress disorder (PTSD) |
| Mengesha and Sarnyai (2025) | Cyclones, droughts, floods, coastal erosion, and sea level rise | Stress, worry, depression, anxiety, posttraumatic stress disorder (PTSD), fatigue, sleep difficulties, intrusive thoughts, sadness, anger, and emotional distress |
| Soutar and Wand (2022) | Climate change awareness and perception / Long-term observed changes | Clinical anxiety, anxiety symptoms (worry, rumination, insomnia, anxiety about future generations, difficulty concentrating), feelings of powerlessness, hopelessness, sadness, solastalgia, and coping strategies (action, support, adaptation, optimism, and hope) |

**Supplementary Table S4.**

*Methodological Quality Assessment of Included Systematic Reviews Using AMSTAR 2*

| Criterion | Gianfredi et al., 2024 | Tito et al., 2024 | Lebel et al. (2022) | Fahrudin et al. (2024) | Vecchio et al. (2022) | Cianconi et al., 2020 | Cosh et al., 2024 | Mengesha and Sarnyai (2025) | Soutar and Wand (2022) |
| --- | --- | --- | --- | --- | --- | --- | --- | --- | --- |
| 1 | Yes | Yes | Yes | Yes | Yes | Yes | Yes | Yes | Yes |
| 2 | Yes | Yes | No | No | No | No | Yes | No | No |
| 3 | Yes | Yes | Yes | Yes | Yes | Yes | Yes | Partial yes | Yes |
| 4 | Yes | Yes | Yes | Yes | Yes | Yes | Yes | Yes | Yes |
| 5 | Yes | Yes | Yes | Yes | Yes | Yes | Yes | Yes | No |
| 6 | Yes | Yes | Yes | No | Yes | No | Yes | Yes | Yes |
| 7 | Yes | Partial yes | Yes | No | Yes | Yes | Yes | No | No |
| 8 | Yes | Yes | Yes | Yes | Yes | Yes | Yes | Yes | Yes |
| 9 | Yes | Yes | No | Yes | Yes | Yes | Yes | Yes | Yes |
| 10 | No | No | No | No | No | No | No | No | No |
| 11 | Not applicable | Not applicable | Not applicable | Not applicable | Not applicable | Not applicable | Not applicable | Not applicable | Not applicable |
| 12 | Not applicable | Not applicable | Not applicable | Not applicable | Not applicable | Not applicable | Not applicable | Not applicable | Not applicable |
| 13 | Yes | Yes | Yes | Partial yes | No | Yes | Yes | Yes | Yes |
| 14 | Yes | Yes | No | Yes | Yes | Yes | Yes | Yes | Yes |
| 15 | Not applicable | Not applicable | Not applicable | Not applicable | Not applicable | Not applicable | Not applicable | Not applicable | Not applicable |
| 16 | Yes | Yes | Yes | Yes | Yes | Yes | Yes | Yes | Yes |
| Overall methodological quality | High | High | Moderate | Critically low | Critically low | Moderate | High | Critically low | Critically low |

*Note.* Critical items: **(2,4,7,9,11,13,15)**

# ****Appendix S1.****

Search strategy

("Climate change" OR "Global warming" OR "Climatic alteration" OR "Climate disruption" OR "Climate variation" OR "Environmental change" OR "Atmospheric change" OR "Climate crisis" OR "Climate shift" OR "Planetary warming") AND ("Psychological impact" OR "Psychosocial impact" OR "Social impact" OR "social effect" OR "Psychological consequence" OR "Psychosocial consequence" OR "Psychological effect" OR "Psychosocial effect" OR "Social and mental impact" OR "Psychosocial influence" OR "Psychological influence" OR "Psychosocial repercussion" OR "Psychological repercussion" OR "Social consequence" OR "Psychosocial outcome" OR "Psychological outcome" OR "Mental Health" OR "Psychological response" OR "substance use disorder" OR "affective disorder " OR "major depressive disorder" OR "persistent depressive disorder" OR "cyclothymia" OR " anxiety disorder " OR "separation anxiety disorder " OR "social anxiety " OR "panic disorder " OR " generalized anxiety disorder " OR " obsessive compulsive disorder " OR " post traumatic stress disorder " OR "PTSD" OR " dissociative disorder "OR "mental health" OR solastalgia OR eco-anxiety OR "environmental distress" OR eco-stress OR eco-guilt OR eco-anguish OR eco-sorrow OR "climate anxiety" OR eco-nostalgia OR eco-fear OR "post-traumatic stress" OR trauma OR eco-depression OR anxiety OR depression OR "complex trauma" OR "anticipatory solastalgia" OR "climate emotions" OR eco-emotions OR stress OR " anxiety disorder " OR " sleep disorder" OR "depression" OR " anxious " OR " suicidality " OR "suicidal ideation" OR "anxiety " OR "psychological resilience" OR "adaptive capacity" OR "Post-traumatic growth" OR "vicarious posttraumatic growth" OR "disasters resilience" OR "climate resilience" OR "climate change resilience") AND ("review" OR "meta-analysis")
